# Comparative Targeted Proteomics of the Central Metabolism and Photosystems in SigE Mutant Strains of *Synechocystis* sp. PCC 6803

**DOI:** 10.3390/molecules23051051

**Published:** 2018-05-01

**Authors:** Yuma Tokumaru, Kiyoka Uebayashi, Masakazu Toyoshima, Takashi Osanai, Fumio Matsuda, Hiroshi Shimizu

**Affiliations:** 1 Department of Bioinformatic Engineering, Graduate School of Information Science and Technology, Osaka University, 1-5 Yamadaoka, Suita, Osaka 565-0871, Japan; yuma_tokumaru@ist.osaka-u.ac.jp (Y.T.); kiyoka_uebayashi@ist.osaka-u.ac.jp (K.U.); toyoshima104@ist.osaka-u.ac.jp (M.T.); fmatsuda@ist.osaka-u.ac.jp (F.M.); 2 School of Agriculture, Meiji University, 1-1-1, Higashimita, Tama, Kawasaki, Kanagawa 214-8571, Japan; tosanai@meiji.ac.jp

**Keywords:** central carbon metabolism, targeted proteome analysis, NAPDH balance, *Synechocystis* sp. PCC 6803, *sigE*

## Abstract

A targeted proteome analysis was conducted to investigate the SigE dependent-regulation of central metabolism in *Synechocystis* sp. PCC 6803 by directly comparing the protein abundance profiles among the wild type, a *sigE* deletion mutant (Δ*sigE*), and a *sigE* over-expression (*sigE*ox) strains. Expression levels of 112 target proteins, including the central metabolism related-enzymes and the subunits of the photosystems, were determined by quantifying the tryptic peptides in the multiple reaction monitoring (MRM) mode of liquid-chromatography–triple quadrupole mass spectrometry (LC–MS/MS). Comparison with gene-expression data showed that although the abundance of Gnd protein was closely correlated with that of *gnd* mRNA, there were poor correlations for GdhA/*gdhA* and glycogen degradation-related genes such as GlgX/*glgX* and GlgP/*glgP* pairs. These results suggested that the regulation of protein translation and degradation played a role in regulating protein abundance. The protein abundance profile suggested that SigE overexpression reduced the proteins involved in photosynthesis and increased GdhA abundance, which is involved in the nitrogen assimilation pathway using NADPH. The results obtained in this study successfully demonstrated that targeted proteome analysis enables direct comparison of the abundance of central metabolism- and photosystem-related proteins.

## 1. Introduction

As a photosynthetic organism, cyanobacteria can be used as a host organism for the direct production of bioenergy and chemicals from solar energy and CO_2_ [1,2,3,4]. A model cyanobacteria, *Synechocystis* sp. PCC 6803, can adapt metabolically to environmental perturbations by modulating protein expression levels [5,6]. A sigma factor, SigE, is a global regulator of central carbon metabolism, whose expression is induced under nitrogen-starved conditions in an NtcA-dependent manner [7,8,9,10,11]. The expression of SigE is also induced at the end of day time or 12 h after light exposure, suggesting that SigE is responsible for a metabolic regulation required to survive in dark conditions [12].

For a detailed investigation of SigE functions, *Synechocystis* sp. PCC 6803 strains lacking (Δ*sigE*) and over-expressing (*sigE*ox) the *sigE* gene have been constructed in previous studies [10,13]. Transcriptome analysis of the Δ*sigE* strain under normal photoautotrophic conditions showed that the expression levels of genes involved in the pentose phosphate pathway (*zwf*, *gnd*, *tal*, and *opcA*), glycolysis (*gap1*, *pfkA* (*sll1196*), and *pyk1*), and glycogen degradation (*glgX* (*slr0237*), *glgP* (*sll1356*)) were reduced by the loss of SigE function [10]. These sugar-metabolism genes that were down-regulated in the Δ*sigE* strain were also down-regulated in ΔsigCDE [14]. It was also reported that *sigE* over-expression in the *sigE*ox strain induced an increase in the expression of genes in the pentose phosphate pathway (*zwf*, *gnd*, *tal*, and *opcA*) and glycogen degradation pathways (*glgX* and *glgP*) [13]. These findings indicated that SigE is a global positive regulator of genes in the pentose phosphate and glycogen degradation pathways. Gene-expression analysis also suggested that the degradation of glycogen into glucose and the regeneration of NADPH by the oxidative pentose phosphate pathway were activated under dark conditions by the function of SigE. NADPH would be used for the maintenance of vital functions in dark conditions instead of being supplied from the photosystem.

A proteome-level analysis is required for a more detailed investigation of the metabolic regulation mechanism. Western blotting analysis of the *sigE*ox strain showed that the expression of enzyme proteins in the oxidative pentose phosphate pathway (Zwf, Gnd) and glycogen degradation pathways (GlgX and GlgP) increased and were consistent with that of gene-expression data [13,15]. A more comprehensive analysis is required because proteome analysis has disclosed the multiple post-transcriptional mechanisms that play important roles in the regulation of protein expression, protein–protein interactions, signaling, and enzymatic activity [16,17,18]. Thus, in this study, the SigE-dependent regulation of central metabolism in *Synechocystis* sp. PCC 6803 was confirmed by the targeted proteome analysis for the direct comparison of the protein abundance profiles among the wild-type (WT), Δ*sigE,* and *sigE*ox strains.

Shotgun proteomics has been employed for the discovery of novel regulation mechanisms in *Synechocystis* sp. PCC 6803 [19,20,21,22,23]. For instance, 45% of *Synechocystis* proteins were simultaneously identified and quantified by shotgun proteomics [20]. Data analysis showed that 155 proteins were differentially expressed across autotrophic, heterotrophic, photoheterotrophic and mixotrophic conditions [20]. On the other hand, targeted proteomics is a method for sensitive, precise quantification of expression levels of preselected proteins [24,25]. In targeted proteomics, a crude protein extract from cyanobacteria cells is digested by trypsin [26]. Expression levels of target proteins are determined by quantifying amounts of the preselected tryptic peptides by the selected or multiple reaction monitoring (MRM) mode of triple quadrupole mass spectrometry. The MRM assay method for cyanobacteria has been comprehensively developed for the central metabolism-related enzymes [27,28]. Targeted proteome analysis using the MRM assay method successfully quantified more proteins than shotgun proteome analysis in *Synechocystis* sp. PCC 6803 grown in iron-deficient conditions [29].

In this study, a targeted proteome analysis was conducted to measure directly the abundance of 144 target proteins in the central metabolism related-enzymes and the photosynthetic apparatus. The protein abundance data, including 112 proteins, were successfully obtained from the WT, Δ*sigE*, and *sigE*ox strains cultured under photoautotrophic conditions to understand the SigE-dependent regulation of central metabolism and photosynthesis at the protein layer. The results showed that one of the most important roles of SigE was as a positive regulator of oxidative pentose phosphate pathway (OxPPP) activity and NADPH reproduction, because the abundances of Gnd in OxPPP and NADPH/NADP^+^ ratios were significantly changed in Δ*sigE* and *sigE*ox. The protein abundance profile also suggested that SigE overexpression increases GdhA abundance, which is involved in the nitrogen assimilation pathway using NADPH and downregulates the proteins involved in photosynthesis. Those results confirmed the SigE-dependent regulation of the C/N balance at the proteome level in *Synechocystis* sp. PCC 6803.

## 2. Results and Discussion

### 2.1. Targeted Proteome Analysis of Wild-Type, sigE Deleted and Overexpressed Strains of Synechocystis sp. PCC 6803

**Figure 1:**
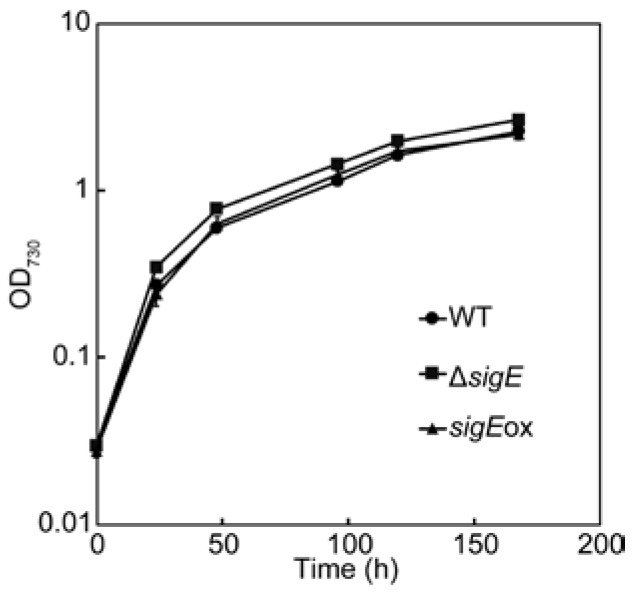
Cell growth curve of wild-type (WT), *sigE* deleted (Δ*sigE*) and overexpressed (*sigE*ox) strains of *Synechocystis* sp. PCC 6803. Flask-scale cultivations were performed in modified BG11 medium containing 5 mM NH_4_Cl as a nitrogen source under photoautotrophic conditions with continuous light at 40 µmol m^−2^ s^−1^. Means of triplicate cultivations were represented with standard deviations.

Three strains of *Synechocystis* sp. PCC 6803, WT, *sigE*-deleted (Δ*sigE*), and overexpressed (*sigE*ox) strains were cultivated in flasks under photoautotrophic conditions with continuous light at 40 µmol m^−2^ s^−1^. Modified BG11 medium containing 5 mM NH_4_Cl as a nitrogen source was employed throughout this study. The culture profile data (Figure 1) showed that cells actively grew until 48 h after the start of cultivation. Although the cell growth rate was reduced after 48 h, cell density was gradually increased and reached an OD_730_ = 2–3 (WT: 2.4, Δ*sigE*: 2.8, and *sigE*ox: 2.3) at 168 h. The growth curves of the three strains including WT, Δ*sigE*, and *sigE*ox were essentially similar to each other, as demonstrated in previous studies [10,13].

For the targeted proteome analysis, crude protein extracts were obtained from cells collected at mid-log phase (OD_730_ = 0.4–0.7). Following reductive alkylation and digestion by trypsin, the tryptic peptide samples were served for the nano liquid-chromatography–triple quadrupole mass spectrometry (LC–MS/MS) analysis. Here, we employed a series of MRM assay methods with 3065 channels to analyze 686 tryptic peptides derived from 144 target proteins including enzyme and subunit proteins responsible for central metabolism and photosynthetic apparatus (Table S1). In order to compare protein levels precisely, fully ^15^N-labeled tryptic peptide samples were prepared from WT, Δ*sigE*, and *sigE*ox strains grown in modified BG11 medium containing ^15^NH_4_Cl as the nitrogen source. The fully ^15^N-labeled tryptic peptide samples were used as internal standards. The tryptic peptides, whose signals were commonly observed among 3 strains in the targeted proteomics data with the largest signal-to-noise ratios, were employed for protein quantification (Table S2). The MRM assays in this study successfully determined the levels of tryptic peptides derived from the 112 proteins (Table S2, Figures S1–S5). Because the signal change of multiple peptides constituting one protein were not significantly different, one of these peptides was selected as a representative for the quantification of protein (Figures S1–S4). 

**Figure 2:**
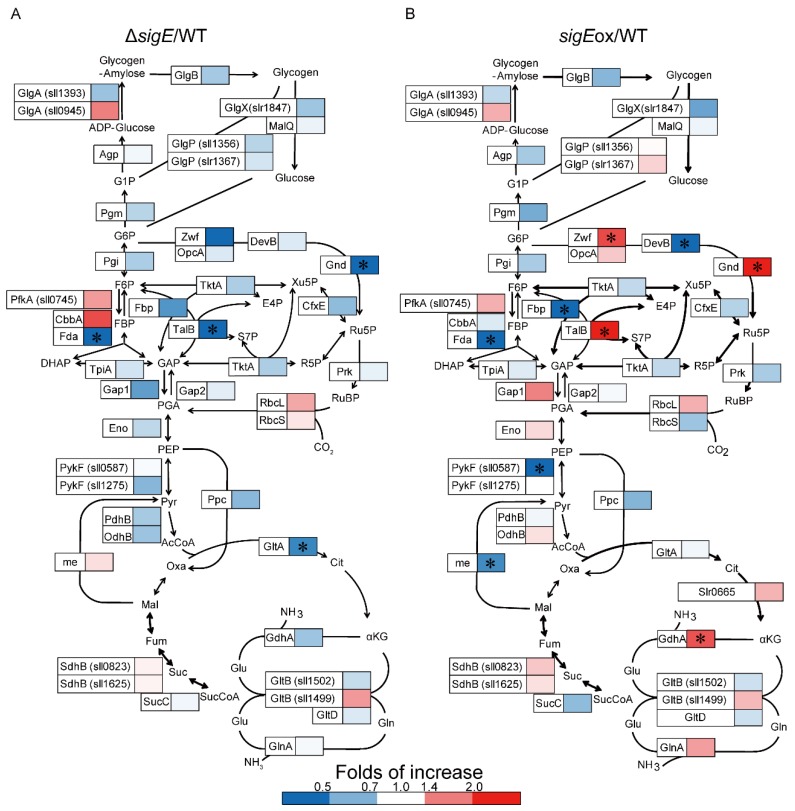
Effect of *sigE* deletion and overexpression on the expression of the central metabolism proteins of *Synechocystis* sp. PCC 6803. Relative expression levels [Δ*sigE*/WT (**A**) and *sigE*ox/WT (**B**)] of proteins determined by the targeted proteome analysis are represented by heat maps. Asterisks indicate a significant difference in terms of both two-sided Student’s *t*-test (α 0.05) and the fold increase (>1.5 and <0.67).

**Figure 3:**
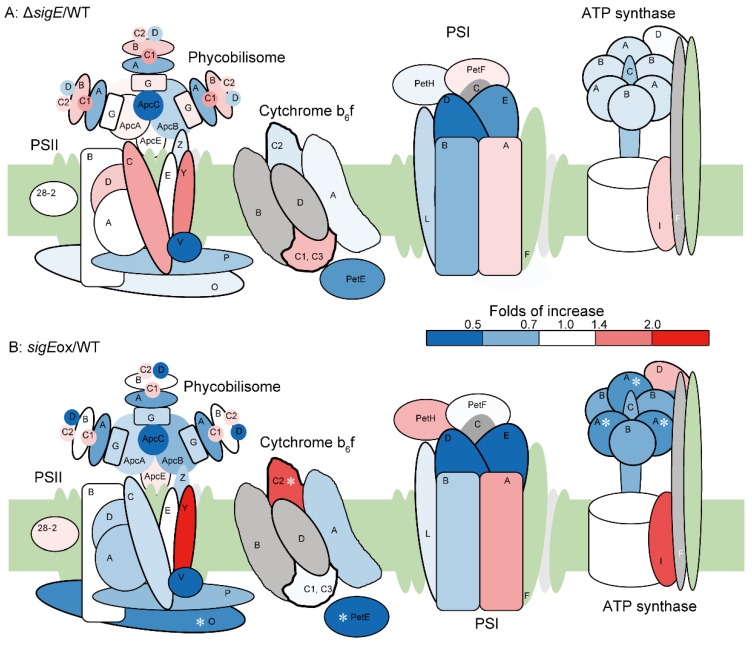
Effect of *sigE* deletion and overexpression on the expression of photosystem-related proteins in *Synechocystis* sp. PCC 6803. Relative expression levels [Δ*sigE*/WT (**A**) and *sigE*ox/WT (**B**)] of proteins determined by the targeted proteome analysis were represented by heat maps. Asterisks indicate a significant difference in terms of both two-sided Student’s *t*-test (α 0.05) and the fold increase (>1.5 and <0.67).

**Figure 4:**
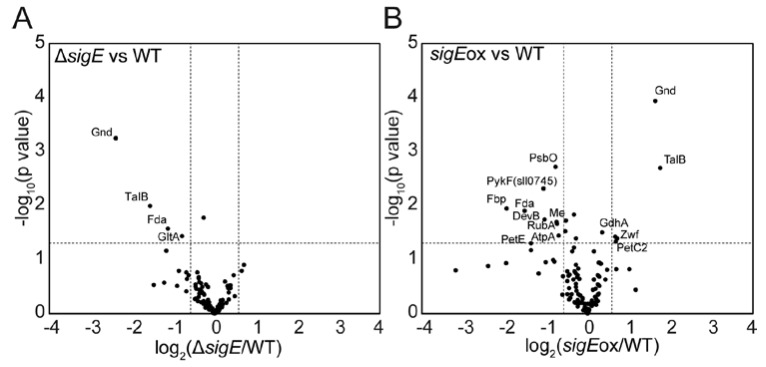
Volcano plot for finding proteins whose abundances were significantly changed in terms of both two-sided Student’s *t*-test (α 0.05) and the fold increase (>1.5 and <0.67). (**A**) Δ*sigE* vs. WT. (**B**) *sigE*ox vs. WT. Values are means of three independent experiments.

Figure 2 and Figure 3 show the heat map representations of the fold change in the relative abundance of central metabolic enzymes (Figure 2) and photosynthetic proteins (Figure 3) in Δ*sigE* (Δ*sigE*/WT) and *sigE*ox (*sigE*ox/WT) compared to those in WT. The enzyme abundance profiles were compared by volcano plots using thresholds with fold of change (FC) > 1.5, FC < 0.667 and *p* value < 0.05 (Figure 4). The comparison of the protein abundance levels of 112 target proteins between WT and Δ*sigE* showed that the abundances of four proteins decreased in Δ*sigE* including Fda, Gnd, TalB, and GltA (Figure 4A). Although it was not statistically significant, the abundance of CbbA increased in Δ*sigE* (Figure 2). Although GltA is an enzyme for citrate synthase in the tricarboxylic acid (TCA) cycle, four proteins including CbbA, Fda, Gnd, and TalB were commonly responsible for the Calvin–Benson cycle and the OxPPP.

The protein abundance profile data showed that two enzymes in the pentose phosphate pathway (PPP), Gnd and TalB, were upregulated in *sigE*ox, whereas abundances of these enzymes were decreased in Δ*sigE* (Figure 2, Figure 4). These results suggested that the expression of Gnd and TalB are positively regulated by SigE. Furthermore, the relative abundances of Fda (Slr0943, fructose-1,6-bisphosphate aldolase, class I) levels decreased in both the Δ*sigE* and *sigE*ox strains while the expression of another aldolase, CbbA (Sll0018, fructose-1,6-bisphosphate aldolase class II), was negatively regulated by SigE (Figure 2, Figure 4). These results suggested that two aldolases, Fda and CbbA, had distinct roles in regulating the Calvin–Benson cycle. Indeed, it has been reported that sedoheptulose-1,7-bisphosphate (SBP) in addition to fructose-1,6-bisphosphate (FBP) could be a substrate of both Fda and CbbA. However, the SBP/FBP activity ratio of the CbbA was two times higher than that of the Fda [30]. The up- and down-regulation of proteins, however, did not affect the normal cell metabolic functions, as the three strains showed similar growth curves (Figure 1).

### 2.2. Comparison with the Gene-Expression Data

**Figure 5:**
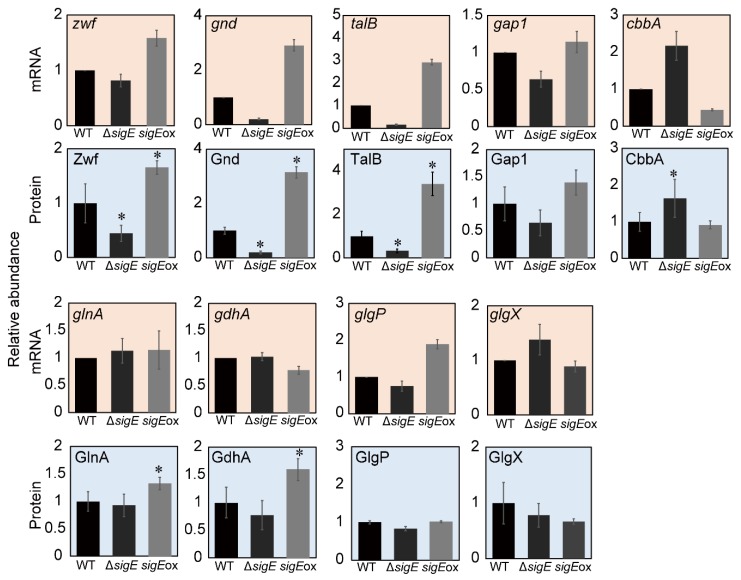
Comparison between gene expression and protein abundance in the wild-type (WT), Δ*sigE*, and *sigE*ox strains. The gene expression data obtained by the microarray analysis was obtained from a previous study [13]. Values are means ± standard deviation (SD) of three independent experiments. Asterisks indicate a significant difference assessed with two-sided Student’s *t*-test with an α level of 0.05.

The relative protein abundance levels determined by the targeted proteome analysis in this study were compared with the gene-expression data obtained by the transcriptome analysis performed in a previous study (Figure 5). As shown in the above section, the abundances of Gnd protein in Δ*sigE* and *sigE*ox strains were significantly lower and higher than that of WT, respectively. Essentially identical patterns were observed for *gnd* mRNA expression levels determined by the previous microarray analysis (Figure 5). The similarities between protein abundance and gene-expression patterns were also observed for Zwf/*zwf* and TalB/*talB* in the PPP, and CbbA/*cbbA*, and Gap1/*gap1* in glycolysis (Figure 5). For instance, protein abundances of GlnA and GdhA in the nitrogen assimilation pathway were different from that of the corresponding genes (Figure 5). Furthermore, the microarray analyses in the previous studies pointed out that the expression levels of glycogen degradation-related genes such as *glgX* (*slr0237*) and *glgP* (*sll1356*) were significantly changed by the regulation of *sigE*. However, this regulation was not observed in protein levels as the relative protein abundances of GlgP and GlgX were different from those of gene expressions (Figure 5). Although this discrepancy may be derived from differences in culture conditions (shaking flask in this study vs. bubbling flask in the previous study), results also suggested that the regulation of protein translation and degradation played a role in regulating protein abundance.

### 2.3. Effect of sigE Deletion and Overexpression on NADPH/NADP^+^ Ratio

The targeted proteome analysis revealed that among the investigated proteins, the abundance of proteins in the PPP, such as Gnd and TalB, were most significantly and directly affected by SigE deletion and overexpression (Figure 2, Figure 4). The direct measurement of protein abundance confirmed that the activation and inactivation of the PPP via Gnd and TalB expression was a key mechanism in the SigE-dependent metabolic regulation in *Synechocystis* sp. PCC 6803.

**Figure 6:**
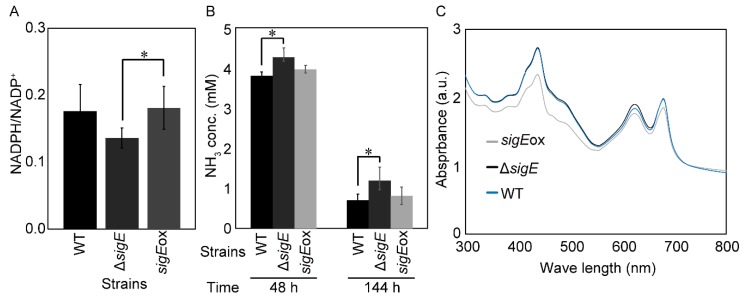
Effect of *sigE* deletion and overexpression on phenotypes of *Synechocystis* sp. PCC 6803. (**A**) NADPH/NADP^+^ ratio. (**B**) NH_3_ concentration in the medium. Values are means ± SD of three independent experiments. Differences were assessed with two-sided Student’s *t*-tests with an alpha level of 0.05. Asterisks indicate a significant difference (*p* < 0.05). (**C**) UV-VIS spectra. Absorbance levels were normalized as ABS730 as 1.0.

Although TalB (transaldolase) is a part of the Calvin–Benson cycle for carbon fixation, Gnd (gluconate-6-phosphate dehydratase) is responsible for NADPH regeneration in the OxPPP. Therefore, the SigE-dependent regulation of Gnd level could affect the NADPH regeneration rate. The NADPH/NADP^+^ assay shown in Figure 6A showed that the NADPH/NADP^+^ ratio in *sigE*ox was 1.33 times larger than that of Δ*sigE*, and that the NADPH/NADP^+^ ratios in Δ*sigE* and *sigE*ox tended to decrease and increase compared to that of wild type, respectively. These results revealed that the abundance of Gnd in the OxPPP was significantly under the control of SigE and that changes in Gnd abundance should affect the NADPH balance via the OxPPP. It has been demonstrated that the metabolic flux level in the OxPPP is up-regulated by treatment with the PSII inhibitor, DCMU [3-(3,4-dichlorophenyl)-1,1-dimethylurea]. In photoheterotrophic conditions, cyanobacteria utilize glucose or glycogen as carbon sources and survive by reproducing NADPH through the OxPPP [31,32].

### 2.4. SigE-Dependent Regulation of the Nitrogen Assimilation Pathway

In cyanobacteria, ammonium is incorporated into 2-oxoglutarate (2-OG) via the glutamine synthetase (GS, GlnA) and glutamate synthase (GOGAT, GltB) cycle, known as the GS–GOGAT pathway [33]. The GS-GOGAT cycle is regulated by the global nitrogen assimilation regulator NtcA. Alternatively, ammonium can be incorporated directly into glutamate by NADP-dependent glutamate dehydrogenase (GdhA), in a less efficient but less energy-consuming manner [34]. The targeted proteome analysis performed in this study showed that the abundance of GdhA was significantly changed in Δ*sigE* strains (Figure 2, Figure 4). The increase in GdhA abundances in *sigE*ox strains should associate with the regulation of the OxPPP as GdhA consumes NADPH for the nitrogen assimilation in *Synechocystis* sp. PCC 6803 [35]. However, increased expression of the *gdhA* gene was not found in the previous transcriptome analysis (Figure 5), suggesting that the regulation of GdhA abundance was sensitive to culture conditions and that protein translation and degradation play a role.

In order to confirm the effect of *sigE* deletion and over-expression on nitrogen assimilation, WT, *sigE*ox, and Δ*sigE* strains were cultivated again under the same conditions to determine concentrations of remaining ammonium in the medium (Figure 6B). The analysis showed that the ammonium concentration of Δ*sigE* at 48 h was 4.3 mM, which was 1.11 times larger than that of WT (Figure 6B). On the other hand, the concentration of remaining ammonium in the *sigE*ox strain was similar to that of WT. Similar results were also observed at 144 h (Figure 6B). Those results suggested that deleting SigE could reduce nitrogen assimilation, probably by decreasing the GS-GOGAT pathway genes such as GdhA abundance and NADPH supply from the oxidative pentose phosphate pathway. 

### 2.5. Protein Abundance Profiles of the Photosynthetic Apparatus 

It was expected that the assimilated nitrogen was mainly used for the biosynthesis of proteins such as the photosynthetic apparatus. The volcano plot of the targeted proteome data (Figure 4) showed that the abundance of PsbO, RubA, AtpA and PetE was significantly decreased in the *sigE*ox strains. PsbO is a manganese-stabilizing polypeptide in PSII that binds to a putative Mn-binding protein and keeps 2 of the 4 Mn-atoms. The absence of PsbO in *Synechocystis* affects the coordination of photosynthesis/respiration [36]. RubA is an iron-sulfur protein (rubredoxin) responsible for the assembly of PSI [37]. AtpA is the alpha subunit of ATP synthase. PetE is a plastocyanin that participates in electron transfer between P700 and the cytochrome b6-f complex in PSI [38]. The abundances of antenna proteins such as ApcA, ApcB and ApcC tended to decrease in the *sigE*ox strain although this effect was not statistically significant.

The comparison of ultraviolet–visible (UV–VIS) spectra among the WT, *sigE*ox, and Δ*sigE* strains revealed that the absorbance spectrum of Δ*sigE* was essentially similar to that of WT (Figure 6C). The result was consistent with the targeted proteome data as the abundance of the photosystem-related proteins was not significantly changed in Δ*sigE*. On the other hand, the absorbance of the carotenoid, the phycocyanin and the chlorophyll in the photosynthetic apparatus (about 550 nm, 630 nm and 680 nm, respectively) was reduced in *sigE*ox, suggesting that photosynthetic apparatus construction was disturbed by SigE overexpression. In particular, the significant decrease of the carotenoid is consistent with the previous studies in which photosynthesis activity was decreased under high light conditions and the activity of non-photochemical quenching (NPQ) was down-regulated in the *sigE*ox strain [15,39]. The decrease of the carotenoid may be related to the fact that metabolic flow was directed to synthesis of the polyhydroxybutyrate (PHB) [15]. However, the photosynthesis activity was maintained under usual light [39]. Accordingly, NADPH/NADP^+^ ratio in *sigE*ox strain was not significantly different from WT strain (Figure 6A). In *sigE*ox strain, PPP and *gdhA* expression were much higher and photosystems expression were lower than in WT, but NADPH/ NADP^+^ ratio, nitrogen assimilation and growth rate were not different. These results indicate that NADPH from PPP reduces the need from photosynthesis.

The down-regulation of photosynthetic apparatus could be explained in the context of the C/N balance [40]. As the photosynthetic apparatus are the most abundant protein complexes in cyanobacteria, it has been considered that antenna proteins could be a nitrogen sink in these species. For instance, phycobilisome and the associated linkers are degraded to supply nitrogen under conditions of N starvation [41]. It was reported that degradation of the nitrogen-rich phycobilisomes starts 12 h after nitrogen starvation [42], and also that reduced CO_2_ fixation leads to the down-regulation of genes encoding proteins involved in nitrogen assimilation [43]. The targeted proteome analysis conducted in this study suggested that *sigE* globally regulated the metabolism to increase nitrogen assimilation in *sigE*ox. These results suggested that the overexpression of *sigE* mimicked the nitrogen-limiting condition in *Synechocystis* cell. This is consistent with in the previous studies, in which *sigE* plays a role in recovery from nitrogen deprivation [44,45,46]. Decreases in the antenna protein would be compensated by improved light reaction efficiency in the photosystem. 

## 3. Materials and Methods

### 3.1. Bacterial Strains and Culture Conditions

*Synechocystis* sp. PCC 6803 GT strain, isolated by Williams [47], the Δ*sigE* strain [10] in which the *sigE* (*sll1689*) gene in the genome was disrupted, and the *sigE*ox strain [13] which constitutively expresses the gene were used in this study. These strains were grown in modified BG11 medium, containing 5 mM NH_4_Cl as a nitrogen source. Cells were grown in 100 mL of medium in 500 mL Erlenmeyer flasks for batch culture under photoautotrophic conditions under continuous light (about 40 µmol m^−2^ s^−1^) and 34 °C, and cells in the linear growth phase (OD_730_ = 0.4–0.7) [48] were collected for analysis. Kanamycin (10 μg mL^−1^) was added in the precultures of Δ*sigE* and *sigE*ox. 

### 3.2. Sample Preparation for Proteome Analysis

Total proteins were extracted as described in Picotti et al. [49]. Cell-culture medium containing cells in the logarithmic growth phase (100 mL, OD_730_ = 0.4–0.7) was collected by centrifugation (5000× *g*, 4 °C, 5 min). Pellets were resuspended in 1 mL of lysis buffer [50 mM HEPES, 15% Glycerol, 15 mM DTT (Dithiothreitol), 100 mM KCl, 5 mM EDTA (Ethylenediamine-*N*,*N*,*N*′,*N*′-tetraacetic acid, disodium salt, dihydrate), and one cOmplete protease inhibitors cocktail (Roche, Basel, Switzerland)]. The suspension was transferred to an Eppendorf tube containing zirconia beads (0.6 and 6 mm beads) and disrupted with a Beads Crusher μT-12 (TAITEC, Saitama, Japan) (3000 min^−1^, 6 min). The resulting solution was centrifuged (15,000 rpm, 4 °C, 5 min), and the supernatant was transferred to a proteomics Eppendorf tube (protein low-adsorption tube) to obtain protein extraction samples. The protein concentration of the extracted sample was measured by the Bradford method, and the total protein amount was adjusted to 50 μg. Denatured buffer (500 mM Tris-HCl, 10 mM EDTA, 7 M Guanidine HCl) was added to the adjusted sample to a total volume of 220 μL.

### 3.3. Reduction and Alkylation/Methanol Chloroform Precipitation

One microliter of 50 mg mL^−1^ DTT was added and shaken at room temperature for 1 h using a tube mixer (CM-1000 Cute Mixer, EYELA, Tokyo, Japan), then 2.5 μL of 50 mg mL^−1^ iodoacetamide (IAA) was added and shaken for 1 h to reduce/alkylate the proteins. Next, the proteins were purified by methanol/chloroform precipitation. Proteins were purified as described in Wessel and Flügge [50]. Cold methanol (600 μL) was added to the sample solution and mixed by inversion, then 150 μL of cold chloroform was added and mixed by inversion. Cold milli-Q water (450 μL) was added and mixed by inversion, followed by centrifugation (15,000 rpm, 4 °C, 5 min). The upper layer was removed, and 450 mL of cold methanol was added and mixed gently by inversion. After centrifuging (15,000 rpm, 4 °C, 5 min) using a swing rotor, the supernatant was removed then additional centrifugation (15,000 rpm, 4 °C, 1 min) was performed to completely remove the supernatant. 

### 3.4. Trypsin/LysC Digestion 

Trypsin hydrolyzed the ester bonds on the carboxyl side of Arg and Lys and LysC on the carboxyl side of Lys. With trypsin alone, Lys which can not be leaved remains, but by combining LysC, the overall degradation efficiency and reproducibility are improved. Trypsin/LysC digestion was performed as described previously [26]. To the supernatant obtained from the above steps, 9 μL of 6 M urea was added and shaken for about 10 min at room temperature using a tube mixer. Thirty-six microliters of 0.1 M Tris-HCl (pH 8.5) was added and ultrasonic treatment and standing on ice were repeated twice for 30 s using an ultrasonic washer (Branson 2510, Danbury, CT, USA) to resuspend the protein precipitate. One microliter of 0.5 mg mL^−1^ LysC solution and 2.5 μL of 1% Protease Max solution ware added and mixed by tapping, then incubated at 25 °C for 3 h. One microliter of 0.5 mg mL^−1^ trypsin solution was added and mixed by tapping, then incubated at 37 °C for 16 h.

### 3.5. Desalting Samples

Sample desalination was performed as described previously [51,52,53]. Milli-Q water (7.5 μL) and 3 μL of 50% formic acid aqueous solution were added to the trypsin digestion product, and the mixture was stirred with a vortex mixer and centrifuged (15,000 rpm, 4 °C, 5 min). Fifty-eight microliters of the supernatant were obtained as a result. In order to carry out relative quantitative analysis, ^15^N samples, obtained by culturing *Synechocystis* sp. PCC 6803 in BG11 medium whose nitrogen source was replaced with ^15^NH_4_Cl, and ^14^N samples were mixed so that the protein amount became 1:1 to prepare an analytical sample. The samples were diluted five times by Reagent A (5% acetonitrile, 0.1% formic acid). The diluted samples were desalted by handmade Stage-tip (3M Empore disk C18). The column was equilibrated with the same bed volume of Reagent B (80% acetonitrile, 0.1% formic acid). Then the column was washed with reagent A by centrifugation (4000× *g*, 10 min, RT). The samples corresponding to 5.8 μg were loaded onto the column, followed by centrifugation (4000× *g*, 10 min, RT). The column was washed by a bed volume of reagent A by centrifugation (4000× *g*, 10 min, RT) twice. The same volume of reagent B was added and centrifuged (4000× *g*, 10 min, RT), and the prepared sample was dried and solidified with a centrifugal concentrator, and then dissolved with 36 µL of 0.1% formic acid before nano LC–MS/MS analysis. 

### 3.6. Design of Multiple Reaction Monitoring (MRM) Assay

First, the target 144 proteins related to the central metabolic pathway and photosynthetic apparatus were selected from the Kyoto Encyclopedia of Genes and Genomes (KEGG) database (http://www.genome.jp/kegg/kegg2.html). The amino acid sequences of target proteins were obtained from Cyanobase (http://genome.microbedb.jp/CyanoBase). The MRM method used to quantify these 144 proteins was created by the open software Skyline version 2.6 [54]. Each protein was subjected to a tryptic peptide filter of 8 to 25 residues and five y-fragments (y1 to y5) were selected for each peptide. Samples of *Synechocystis* sp. PCC 6803 were analyzed once by nano LC–MS/MS (LCMS-8060, Shimadzu, Kyoto, Japan) by the provisional MRM method. From the results obtained by the analysis, peak picking was performed based on the shape, coelution, and intensity of the peak, and the best transitions up to 5 were selected, respectively. For proteins with no suitable tryptic peptides and transitions, transitions were quantified for all y fragments and b fragments, from which the tryptic peptides suitable for quantitation were selected again to create the final MRM method.

### 3.7. Nano Liquid-Chromatography–Triple Quadrupole Mass Spectrometry (LC–MS/MS) Analysis by MRM Assay

The trypsin-digested samples were analyzed by a quadrupole mass spectrometer (LCMS-8060, Shimadzu) as described previously [55]. Electrospray ionization (ESI) was performed, and sample separation was performed by nanoLC (LC-20ADnano, Shimadzu). The analytical conditions were as follows: high-performance liquid chromatography (HPLC) column, l-column ODS (pore size: 5 μm, 0.1 × 150 mm, CERI, Tokyo, Japan); trap column, l-column ODS (pore size: 5 μm, 0.3 × 5 mm, CERI); solvent system, water (0.1% formic acid) : acetonitrile (0.1% formic acid); gradient program, 10:90, *v*/*v* at 0 min, 10:90 at 10 min, 40:60 at 45 min, 95:5 at 55 min, and 90:10 at 65 min; and flow rate, 400 nL min^−1^. Mass spectrometry was performed in MRM mode, ESI was 1.6 kV, capillary temperature was 150 °C, collision gas was 270 kPa, resolution of Q 1 and Q 3 was Low, dwell time was 1.0 ms, pause time was 1.0 ms, retention time window was 2 min. One tryptic peptide was selected for each protein and quantified by the peak area ratio of ^14^N sample to ^15^N sample. 

### 3.8. Quantification of NH_3_ in the Medium

The extracellular NH_3_ assay was performed by F-kit (JK International) according to the manufacturer’s protocol. Briefly, the medium was centrifuged (15,000 rpm, 4 °C, 1 min) and the supernatant was obtained, followed by heating at 80 °C for 20 min to inactivate remaining enzyme. Reagent Mix (200 μL) was reacted with 6.7 μL of the sample and incubated. A_340_ was measured after 5 and 20 min. 

### 3.9. Ultraviolet–Visible (UV–VIS) Spectrum

A DU 8000 was used to measure the UV–VIS spectrum. Absorption spectra of cell suspensions were measured according to the ‘opal glass method,’ with a translucent cuvette placed in front of the detector to minimize the effect of light scattering [56]. The results obtained were normalized to the absorbance at 730 nm of chlorophyll as 1.0.

## 4. Conclusions

Targeted proteome analysis was conducted in this study to directly compare the abundance of the central metabolism- and the photosystem-related proteins among WT, Δ*sigE,* and *sigE*ox strains of *Synechocystis* sp. PCC 6803. The analysis showed the SigE-dependent regulation of central metabolism at the protein abundance level. Among the investigated proteins, the most direct or tight regulation via gene expression was observed for the proteins in the pentose phosphate pathway such as TalB and Gnd. Further investigations of protein abundance and their modification states such as phosphorylation by proteomic analysis should uncover the detailed regulatory mechanism of central carbon metabolism in cyanobacteria.

## Supplementary Materials

The following are available online, Figure S1: Selected reaction monitoring (SRM) spectra of the peptide EVTASLVGADAGK (ApcB), Figure S2: SRM spectra of the peptide SYFASGELR (ApcB), Figure S3: SRM spectra of the peptide AVLPQNLTQAQR (Gnd), Figure S4: SRM spectra of the peptide ELEPILTK (Gnd), Figure S5: SRM spectra of the peptide VPATIEEIAAR (Eno), Figure S6: Comparison with the relative abundances of mRNAs and proteins, Table S1: MRM assay method used in this study, Table S2: Targeted proteomics data; Table S3: All analyzed peptides.
