# Supplementary material for: Comparative Targeted Proteomics of the Central Metabolism and Photosystems in SigE Mutant Strains of Synechocystis sp. PCC 6803
Source: Molecules. 2018 May 1;23(5):1051. doi: 10.3390/molecules23051051 (PMC6102573; doi:10.3390/molecules23051051)
Supplement: Supplementary file 1 [file molecules-23-01051-s001.zip › FigureS1_6.pdf]

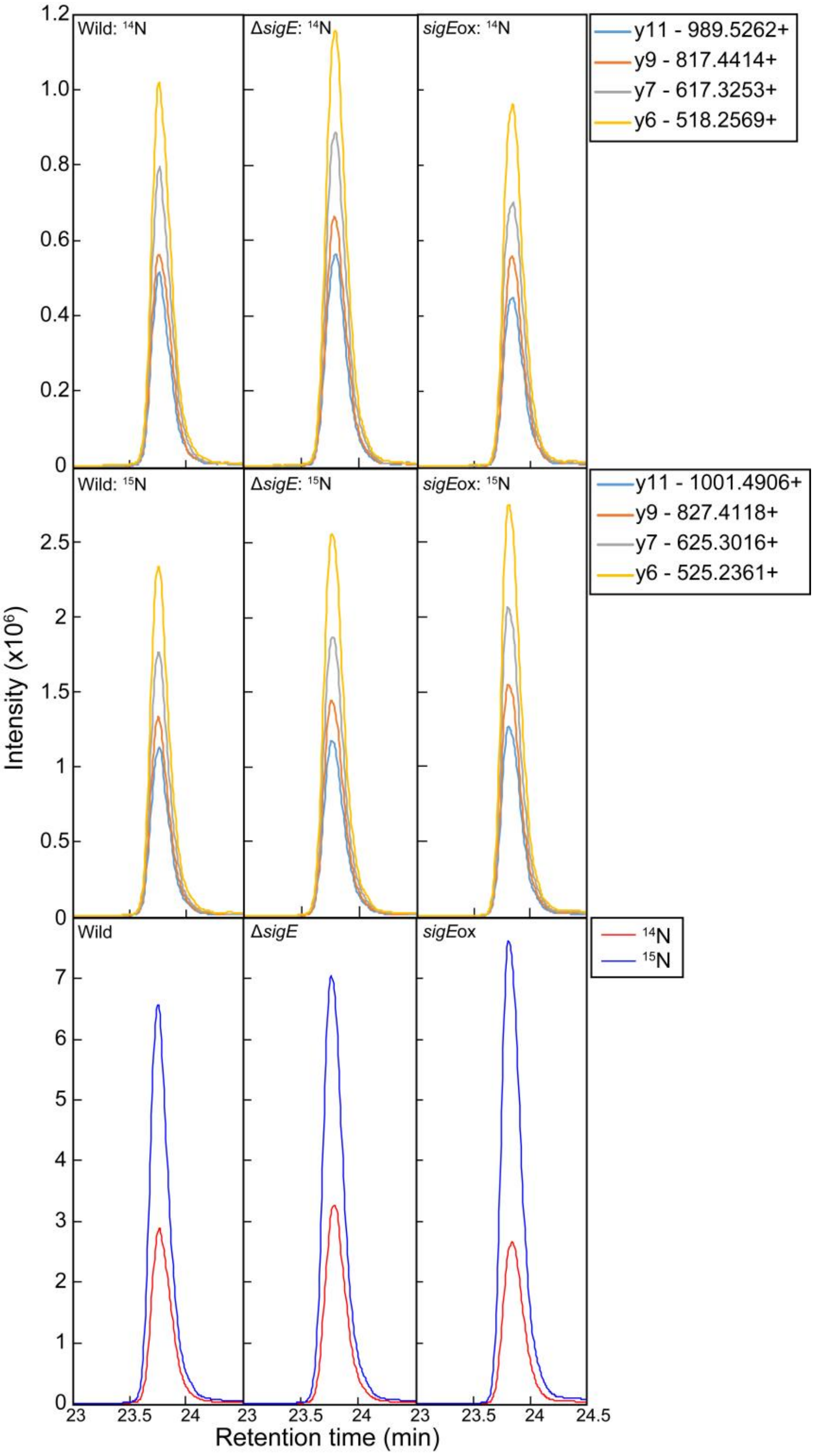

Figure S1 SRM spectra of the peptide EVTASLVGADAGK (ApcB). (Top) Transition spectra of ^{14}N peptide sample. (Middle) Transition spectra of ^{15}N-labeled peptide sample. (Bottom) ^{15}N-labeled peptide and ^{14}N peptide spectra.

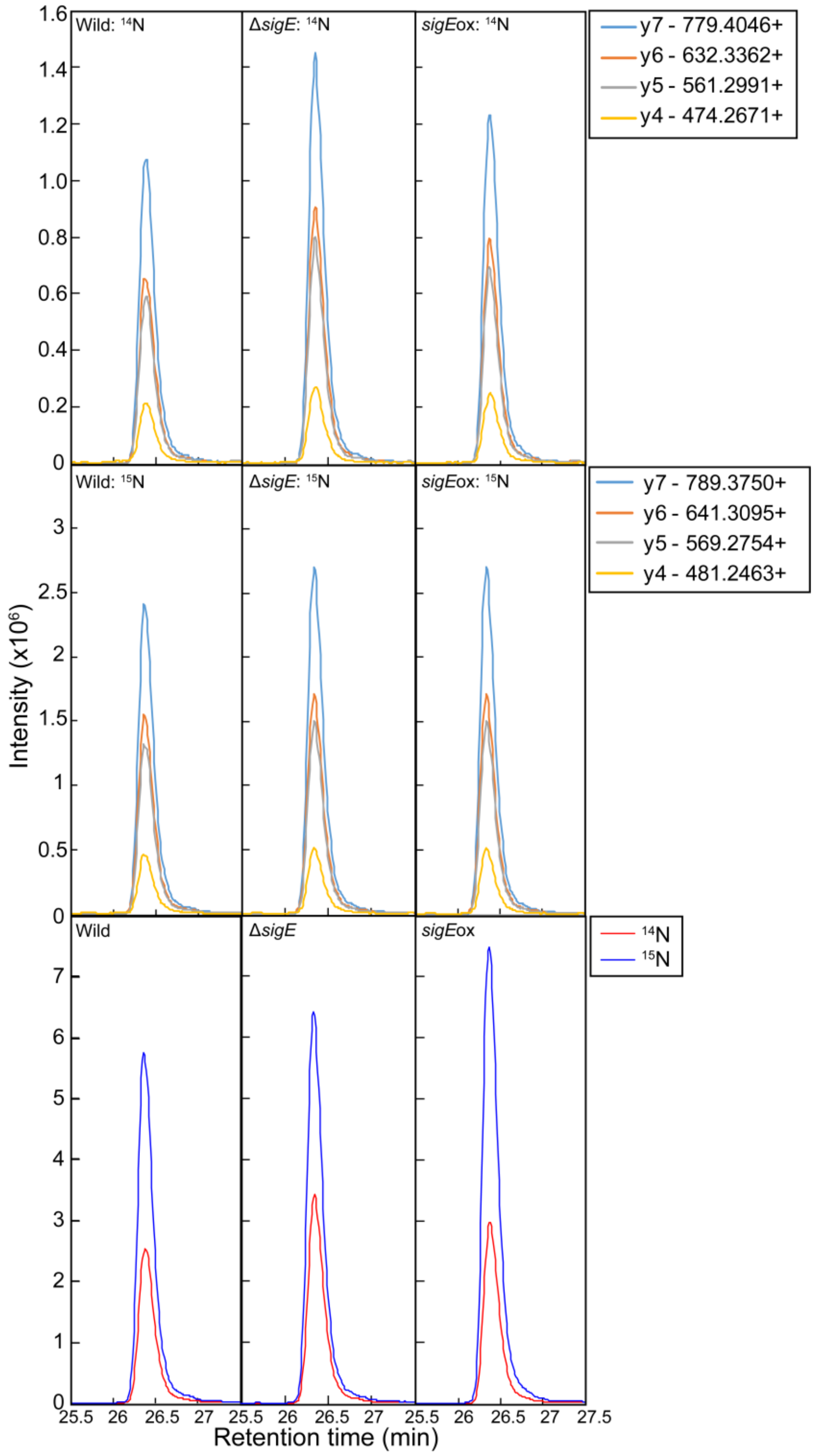

Figure S2 SRM spectra of the peptide SYFASGELR (ApcB). (Top) Transition spectra of ^{14}N peptide sample. (Middle) Transition spectra of ^{15}N-labeled peptide sample. (Bottom) ^{15}N-labeled peptide and ^{14}N peptide spectra.

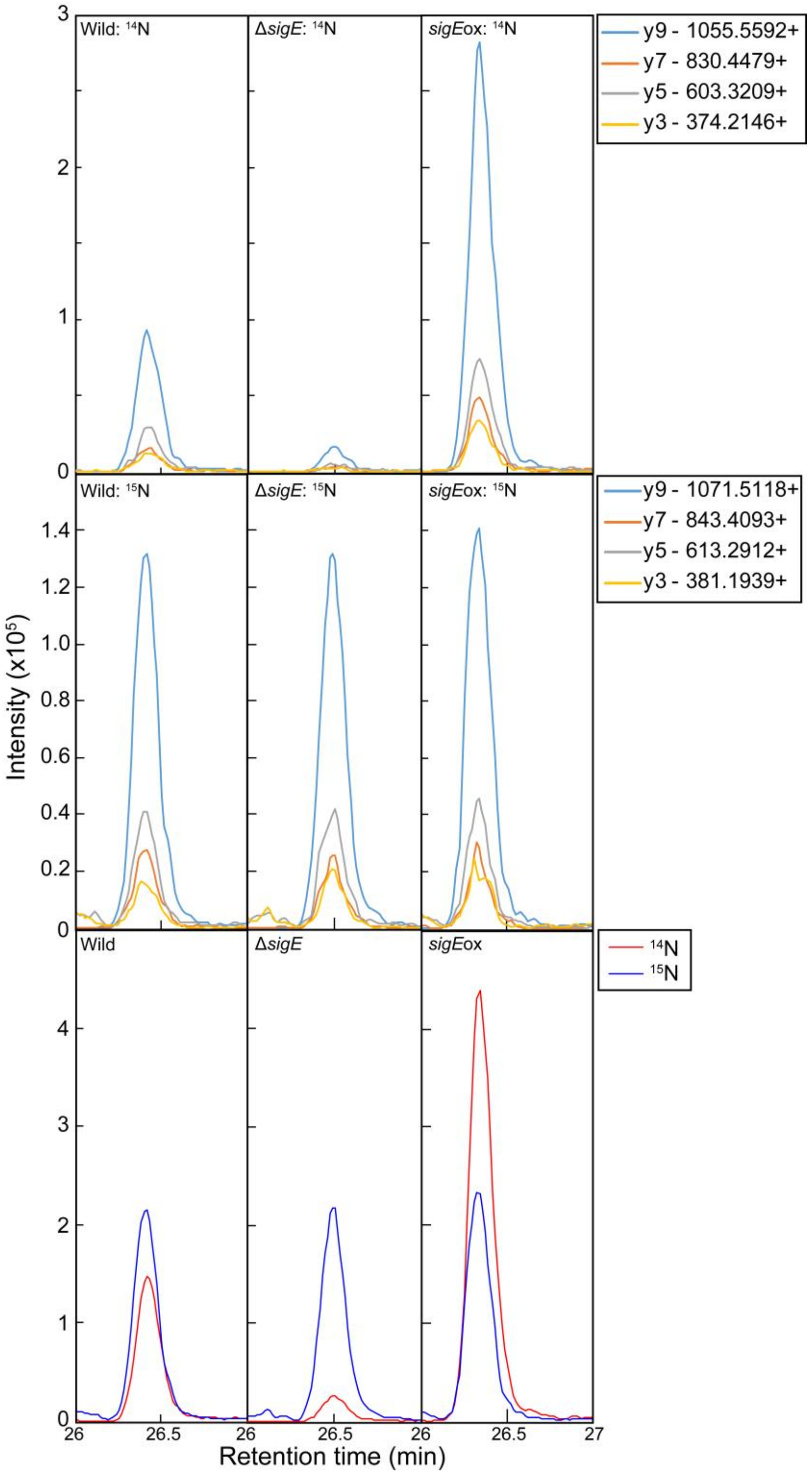

Figure S3 SRM spectra of the peptide AVLPQNLTQAQR (Gnd). (Top) Transition spectra of ^{14}N peptide sample. (Middle) Transition spectra of ^{15}N-labeled peptide sample. (Bottom) ^{15}N-labeled peptide and ^{14}N peptide spectra.

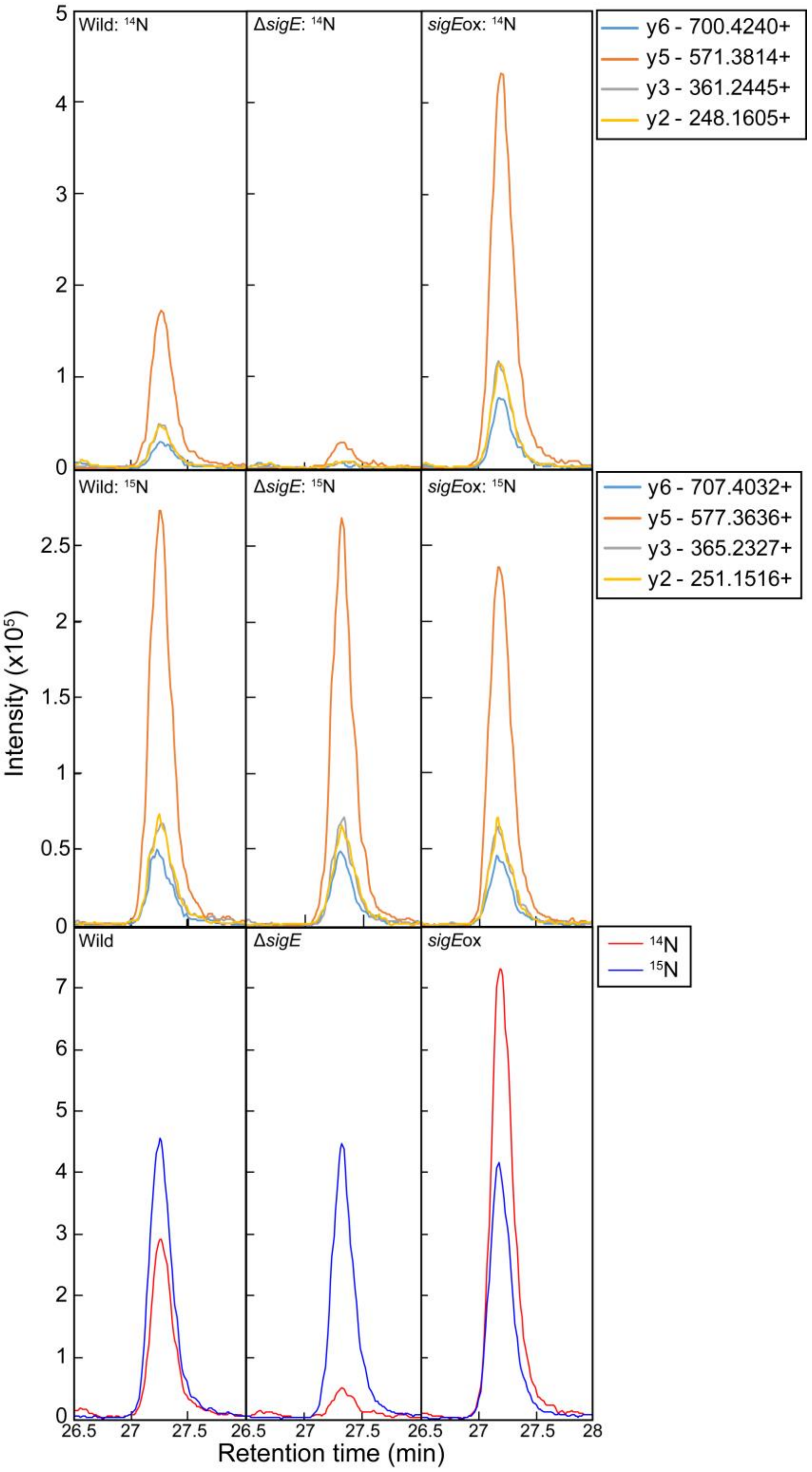

Figure S4 SRM spectra of the peptide ELEPILTK (Gnd). (Top) Transition spectra of ^{14}N peptide sample. (Middle) Transition spectra of ^{15}N-labeled peptide sample. (Bottom) ^{15}N-labeled peptide and ^{14}N peptide spectra.

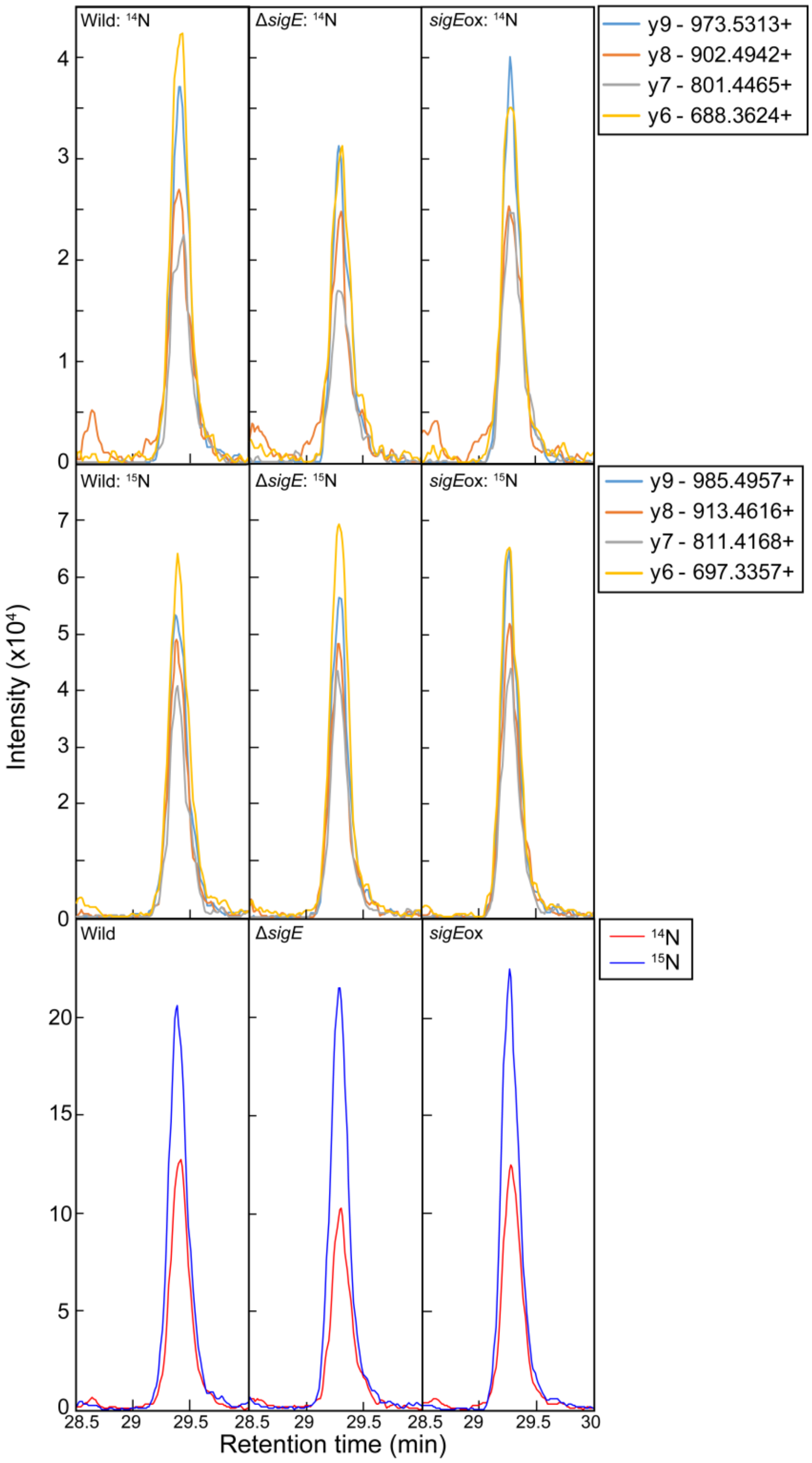

Figure S5 SRM spectra of the peptide VPATIEEIAAR (Eno). (Top) Transition spectra of ^{14}N peptide sample. (Middle) Transition spectra of ^{15}N-labeled peptide sample. (Bottom) ^{15}N-labeled peptide and ^{14}N peptide spectra.

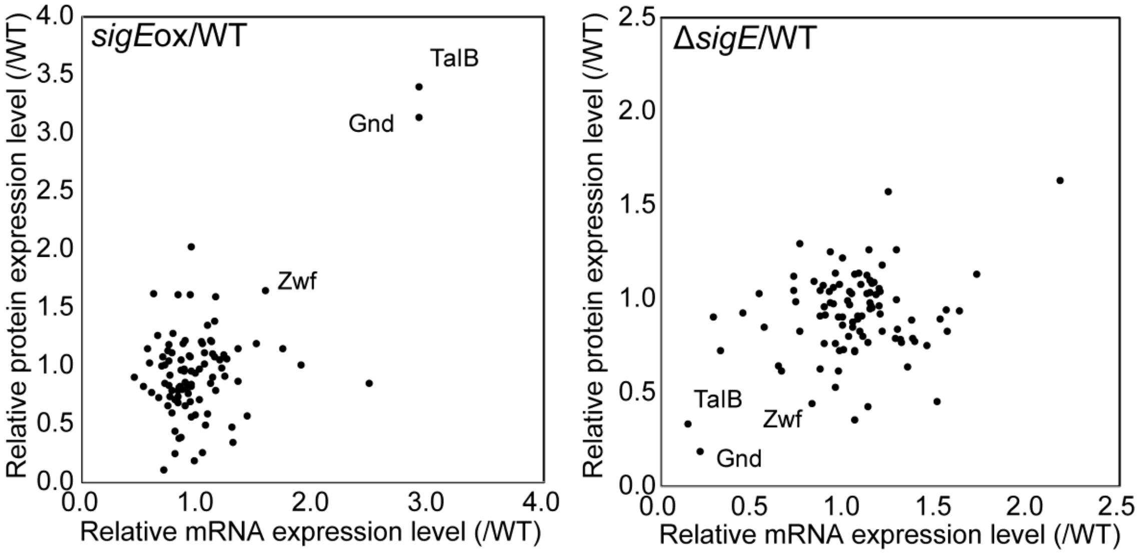

Figure S6 Comparison with the relative abundances of mRNAs and proteins
